# Osmotic Stress Changes the Expression and Subcellular Localization of the Batten Disease Protein CLN3

**DOI:** 10.1371/journal.pone.0066203

**Published:** 2013-06-20

**Authors:** Amanda Getty, Attila D. Kovács, Tímea Lengyel-Nelson, Andrew Cardillo, Caitlin Hof, Chun-Hung Chan, David A. Pearce

**Affiliations:** 1 Sanford Children's Health Research Center, Sanford Research/USD, Sioux Falls, South Dakota, United States of America; 2 Department of Pediatrics, Sanford School of Medicine, University of South Dakota, Sioux Falls, South Dakota, United States of America; Weizmann Institute of Science, Israel

## Abstract

Juvenile CLN3 disease (formerly known as juvenile neuronal ceroid lipofuscinosis) is a fatal childhood neurodegenerative disorder caused by mutations in the *CLN3* gene. *CLN3* encodes a putative lysosomal transmembrane protein with unknown function. Previous cell culture studies using CLN3-overexpressing vectors and/or anti-CLN3 antibodies with questionable specificity have also localized CLN3 in cellular structures other than lysosomes. Osmoregulation of the mouse *Cln3* mRNA level in kidney cells was recently reported. To clarify the subcellular localization of the CLN3 protein and to investigate if human CLN3 expression and localization is affected by osmotic changes we generated a stably transfected BHK (baby hamster kidney) cell line that expresses a moderate level of myc-tagged human CLN3 under the control of the human ubiquitin C promoter. Hyperosmolarity (800 mOsm), achieved by either NaCl/urea or sucrose, dramatically increased the mRNA and protein levels of CLN3 as determined by quantitative real-time PCR and Western blotting. Under isotonic conditions (300 mOsm), human CLN3 was found in a punctate vesicular pattern surrounding the nucleus with prominent Golgi and lysosomal localizations. CLN3-positive early endosomes, late endosomes and cholesterol/sphingolipid-enriched plasma membrane microdomain caveolae were also observed. Increasing the osmolarity of the culture medium to 800 mOsm extended CLN3 distribution away from the perinuclear region and enhanced the lysosomal localization of CLN3. Our results reveal that CLN3 has multiple subcellular localizations within the cell, which, together with its expression, prominently change following osmotic stress. These data suggest that CLN3 is involved in the response and adaptation to cellular stress.

## Introduction

Mutations of the *CLN3* gene cause juvenile CLN3 disease [1], [2] (formerly known as juvenile neuronal ceroid lipofuscinosis [3] or juvenile Batten disease [4]). Development of treatments for this fatal, childhood neurodegenerative disease is difficult because the primary function of the CLN3 protein is not known, yet. Cells from juvenile CLN3 disease patients, cells derived from CLN3-deficient mouse models and genetic deletions in model systems have been studied and have revealed cellular consequences of the loss of CLN3. Characterization of the primary function of CLN3, however, has been challenging. No common folds or sequences align the CLN3 amino acid sequence with other protein families. CLN3 is an integral membrane protein with six transmembrane domains [5]. The N- and C-termini are both found in the cytosol, as determined by selective permeability experiments [6], [7]. Two glycosylation sites have been confirmed biochemically at asparagines (N-71 and N-85) [8]. The C-terminus is farnesylated [9], [10], and this modification was found to be important for CLN3 localization in one study [8]. There are three lysosomal localization motifs in the sequence of CLN3: two dileucine sorting motifs in the cytosolic internal loop, and an acidic patch found in the C-terminus [11]–[13]. CLN3 may also be phosphorylated at serine and threonine residues [14]–[16].

Most often, CLN3 has been localized to the lysosomal compartment [6]–[8], [17]–[20]. However, CLN3 has also been found in the Golgi [21], [22], endosomes [6], [7], [21], in lipid rafts [21], and at the plasma membrane [17], [21], [23]. In neurons, the protein has been found at synapses and down neuronal processes [14], [19], [24]. The above studies, however, used CLN3-overexpressing vectors and/or anti-CLN3 antibodies with questionable specificity. Only in one study was the CLN3-specific antibody validated by mass-spectrometry [6], and this antibody was not made commercially available. To date, all available CLN3 antibodies have been examined systematically in our lab and no antibodies have specificity when immunoblotting for CLN3 at endogenous levels in wild-type mouse tissue using *Cln3^−/−^* mouse tissue as a negative control (our unpublished results). Therefore, in order to study CLN3 biology in the cell, tagging CLN3 with a detectible epitope is required. In previous studies, exogenous expression of CLN3 has been most often driven by the CMV (cytomegalovirus) promoter, which causes high overexpression, and may therefore introduce artifacts. There is much to be gained by studying the cell biology of CLN3 with these less than optimal conditions, but expression and detection must be optimized to minimize deviation from physiological conditions.

Here we describe an expression vector designed to study detectible CLN3 in several cell types in order to potentially determine conditional regulation or function of CLN3. The human ubiquitin C promoter was chosen to drive stable CLN3 expression at a moderate but probably higher than endogenous level [25]. Additionally, a myc epitope tag was N-terminally added to CLN3 to detect the protein specifically. Since osmoregulation of the mouse *Cln3* mRNA level in kidney cells was recently reported [26], we generated a BHK (baby hamster kidney) clonal cell line that stably expresses myc-tagged human CLN3, and studied the expression and subcellular localization of CLN3 under isotonic and hyperosmotic conditions.

## Results

### Generation of a BHK cell line stably expressing myc-tagged human CLN3

The human *CLN3* cDNA was cloned into the pUB6-B expression vector that utilizes the human ubiquitin C promoter. An N-terminal myc-tag was added and a stop codon was included at the C-terminus to prevent addition of a C-terminal 6-His-tag. The modified cDNA was cloned in-frame in the vector at the *Xho1* and *Age1* restriction sites to omit the C-terminal V5 epitope tag. Proper ligation of the resulting plasmid was confirmed by *HindIII* diagnostic digest and DNA sequencing. The resulting UB6-myc-CLN3 plasmid was used in subsequent transfections, with the UB6 empty vector used as a negative control. The pUB6-B expression vector provides resistance against the antibiotic, blasticidin, and thus, stable transfectants can be selected in blasticidin-containing medium.

**Figure 1:**
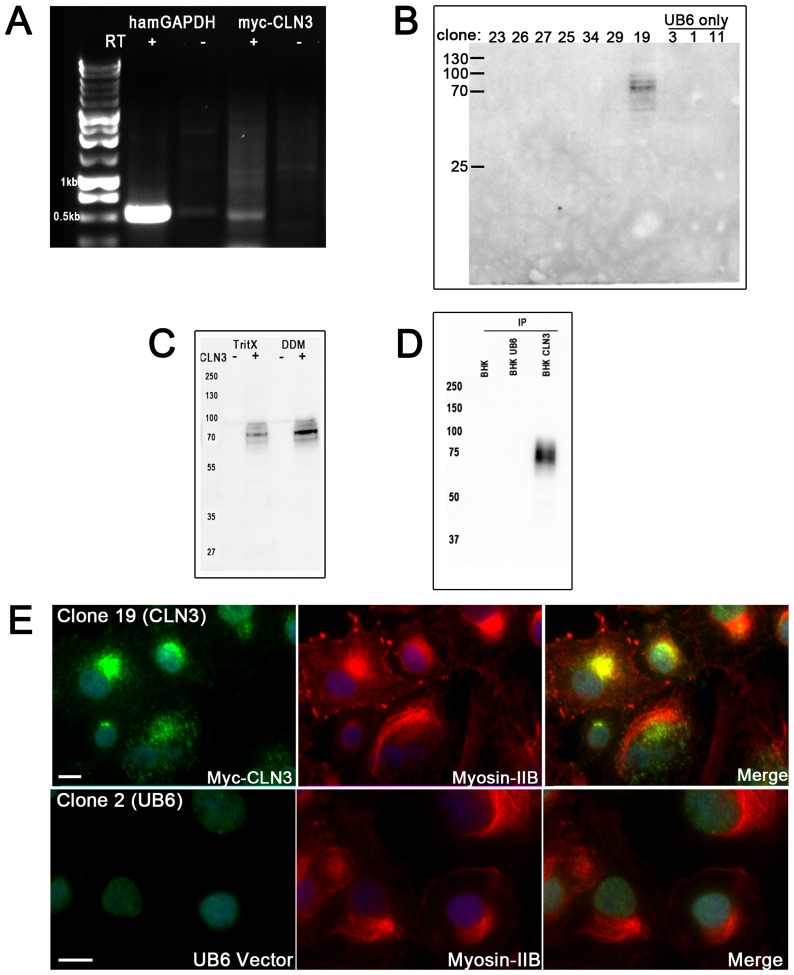
Identification of BHK clone 19 as stably expressing myc-CLN3. BHK clones were screened for stable expression of myc-CLN3. (**A**) A myc-CLN3 positive clone was identified by mRNA expression, with hamster GAPDH and reverse transcriptase (RT) (+/–) controls. (**B**) Immunoblotting of cell lysates from candidate clones shows detectable myc-CLN3 expression in clone 19. Lysates were prepared using 1% DDM detergent in a phosphate buffer. Thirtyµg of protein extract from each clone was run on a 10% polyacrylamide gel and immunoblotted with a monoclonal anti-myc antibody. Clones 23, 26, 27, 25, 34 and 29 did not have detectible myc-CLN3 expression. Clones 3, 1 and 11 were control BHK cells stably expressing the UB6 empty vector only, selected by blasticidin resistance. Clone 19 was the only clone that expressed detectible protein levels of myc-CLN3. (**C**) Lysis buffer containing the detergent, n-Dodecyl β-D-maltoside (DDM; 1%), extracts myc-CLN3 more efficiently than lysis buffer containing Triton X-100 (TritX; 1%). Myc-CLN3 protein was detected by immunoblot using a monoclonal anti-myc antibody. (**D**) In cell lysates prepared with 1% DDM detergent, myc-CLN3 can be immunoprecipitated using a polyclonal (rabbit) anti-myc antibody, and immunoblotted with a monoclonal (mouse) anti-myc antibody. The myc-tag is accessible for Western blotting and for immunoprecipitation from cell lysates. (**E**) Myc-CLN3 has perinuclear localization and partly colocalizes with its recently described interaction partner, myosin IIB. BHK clone 19 cells were examined by immunocytochemistry with antibodies against the myc-tag (green) and myosin IIB (red), and staining the nuclei with DAPI (blue). Only weak nuclear staining (endogenous c-myc) was observed with the anti-myc antibody in a blasticidin-resistant clone that was transfected with the empty UB6 vector (UB6 clone 2). In the merged images yellow indicate the colocalization of myc-CLN3 and myosin IIB. Images were acquired with a confocal microscope. Control BHK cells stably expressing the UB6 vector only show nonspecific staining similar to secondary antibody only controls. Scale bars indicate 10 µm.

Identification and validation of useful clones were completed by RT-PCR and immunoblotting. BHK clone 19 was identified to express *myc-CLN3* mRNA (Figure 1A). Screening several blasticidin-resistant BHK clones by immunoblotting also revealed clone 19 as positive for myc-CLN3 protein expression by a band at ∼70 kDa (Figure 1B). It was confirmed later that this is a glycosylated form of myc-CLN3. Lysis buffer containing the detergent, n-Dodecyl β-D-maltoside (DDM; 1%), extracted myc-CLN3 more efficiently than lysis buffer containing Triton X-100 (1%) (Figure 1C). In cell lysates prepared with 1% DDM detergent, myc-CLN3 could be immunoprecipitated using a polyclonal (rabbit) anti-myc antibody, and immunoblotted with a monoclonal (mouse) anti-myc antibody (Figure 1D), which indicated that the N-terminal myc-tag is available for immunoprecipitation under non-denaturing conditions. Immunofluorescent staining of BHK clone 19 cells with the polyclonal myc-specific antibody revealed a perinuclear and punctate staining for myc-CLN3 (Figure 1E). Only weak nuclear staining (endogenous c-myc) was observed with the anti-myc antibody in a blasticidin-resistant clone that was transfected with the empty UB6 vector (UB6 clone 2; Figure 1E). In clone 19 cells, myc-CLN3 partly colocalized with its recently described interactor, myosin IIB (Figure 1E).

### Osmotic stress upregulates CLN3 expression

**Figure 2:**
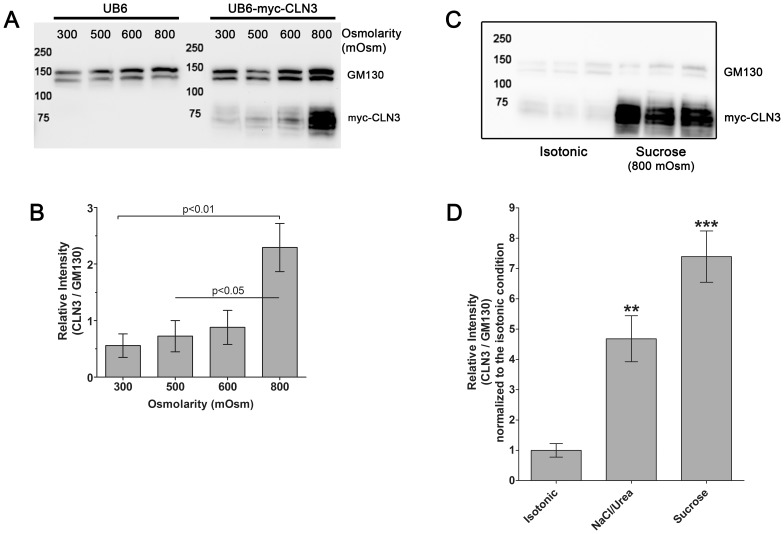
Increased osmolarity upregulates myc-CLN3 protein expression. (**A**) Hyperosmolarity induced by NaCl/urea dramatically increases myc-CLN3 protein expression. BHK clone 19 myc-CLN3-expressing and BHK clone 2 UB6 empty vector-expressing cells were grown under isotonic (300 mOsm) or hyperosmotic conditions. Osmolarity was increased at 100 mOsm intervals to 500, 600 or 800 mOsm by the addition of NaCl plus urea (1.5:1 molar ratio). After being exposed to 500, 600 or 800 mOsm for 24 hours, cell lysates were prepared using 1% DDM detergent under non-denaturing conditions. Twenty-five-µg protein from each sample was loaded on a 10% polyacrylamide gel and immunoblotted with a monoclonal anti-myc antibody. GM130, an integral Golgi membrane protein (130 kDa) was immunoblotted as a loading control. The immunoblot (**A**) is representative of 4 biological replicates. (**B**) Densitometric quantification of myc-CLN3 expression in hyperosmolarity induced by NaCl/urea. The mean pixel density for each band was measured in the ImageJ program. Myc-CLN3 band intensities were normalized to the corresponding GM130 (loading control) band intensities. Columns and bars represent mean ± S.E.M. (n = 4). Statistical significance was determined by one-way ANOVA with Bonferroni’s post-test. **(C)** Hyperosmolarity induced by sucrose also significantly increases myc-CLN3 protein expression. BHK clone 19 myc-CLN3-expressing cells were grown under isotonic (300 mOsm) or hyperosmotic conditions. Osmolarity was increased by sucrose at 100 mOsm intervals to 800 mOsm. Cell lysis and the immunoblot for myc-CLN3 were performed as described in (A). (**D**) Densitometric quantification of myc-CLN3 expression in hyperosmolarity induced by sucrose. Myc-CLN3 band intensities were normalized to the corresponding GM130 (loading control) band intensities, and expressed as fold increase of the isotonic control. Columns and bars represent mean ± S.E.M. (n = 3–4). The myc-CLN3 protein level observed when hyperosmolarity was induced by NaCl/urea (B) is shown for comparison. Statistical significance was determined by one-way ANOVA with Bonferroni’s post-test: **p<0.01 and ***p<0.001 as compared to the isotonic control.

**Figure 3:**
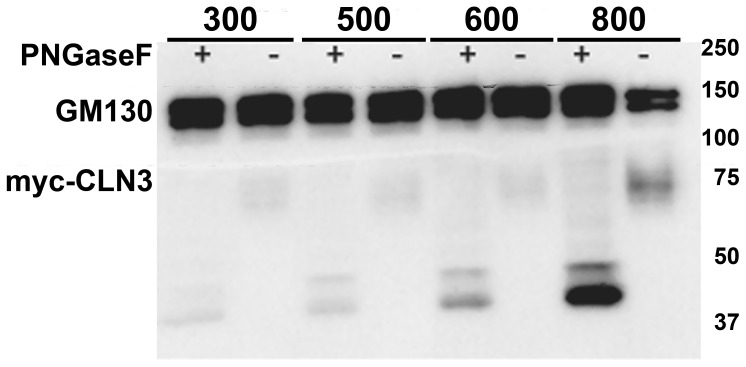
Glycosylation of CLN3 does not appear affected under increasing osmolarity. BHK clone 19 myc-CLN3-expressing cells were grown under isotonic (300 mOsm) or hyperosmotic conditions. Osmolarity was increased at 100 mOsm intervals to 500, 600 or 800 mOsm by the addition of NaCl plus urea (1.5∶1 molar ratio). After being exposed to 500, 600 or 800 mOsm for 24 hours, cell lysates were prepared using 1% DDM detergent under non-denaturing conditions. Twenty-five-µg protein from each sample was treated with the N-glycosylase, PNGase F. PNGase F-treated and untreated protein samples were loaded on a 10% polyacrylamide gel and immunoblotted with a monoclonal anti-myc antibody. GM130, an integral Golgi membrane protein of 130 kDa was immunoblotted as a loading control. Treatment with PNGase F resulted in the same lower molecular weight bands (40–45 kDa) at 300, 500, 600 and 800 mOsm.

As *Cln3* has been proposed to have important roles in the mouse kidney [26], the protein level of myc-CLN3 was examined under increasing osmolarity. In these experiments, the myc-CLN3 expression is driven by the exogenous human ubiquitin C promoter, therefore osmoregulation of the myc-CLN3 protein could be observed only at the translational level. The protein level of myc-CLN3 was examined in BHK (baby hamster kidney) clone 19 myc-CLN3-expressing and BHK clone 2 UB6 empty vector-expressing cells grown under isotonic (300 mOsm) or hyperosmotic conditions. Osmolarity was increased at 100 mOsm intervals to 500, 600 or 800 mOsm by the addition of NaCl plus urea (1.5∶1 molar ratio). These are physiologically relevant values since in the kidney medulla osmolality can exceed 1200 mOsm/kgH_2_O [27]. After being exposed to 500, 600 or 800 mOsm for 24 hours, BHK cell lysates were prepared using 1% DDM detergent-containing nondenaturing lysis buffer and were immunoblotted for the myc-tag and GM130, an integral Golgi membrane protein, as a loading control. As expected no myc-CLN3 expression was detected in BHK clone 2 UB6 empty vector-expressing cells (Figure 2A). In BHK clone 19 myc-CLN3-expressing cells, increasing osmolarity to 800 mOsm dramatically increased myc-CLN3 protein expression (Figure 2A). Densitometric analysis of the relative intensity of myc-CLN3 to GM130 revealed a more than 4-fold increase of myc-CLN3 protein level at 800 mOsm (Figure 2B). Since CLN3 is a glycosylated protein, we examined if increasing osmolarity changes its glycosylation. Treatment with the N-glycosylase, PNGase F, resulted in the same lower molecular weight bands at 300, 500, 600 and 800 mOsm (Figure 3), indicating that glycosylation of CLN3 is not affected by osmotic stress.

CLN3 has been shown to interact with the plasma membrane Na^+^, K^+^-ATPase [28], and therefore this interaction may be required to deal with the increased Na^+^ influx at high concentrations of NaCl in the culture medium. In order to determine if myc-CLN3 expression is responsive to hyperosmotic stress in general, cells were treated with sucrose as an osmolyte. Sucrose is impermeable to mammalian cells, and therefore, its effect is purely osmotic. Osmolarity was increased by sucrose at 100 mOsm intervals to 800 mOsm. After being exposed to 800 mOsm for 24 hours, cell lysates were examined for myc-CLN3 expression. The osmotic stress induced by sucrose also remarkably increased the protein level of myc-CLN3 (Figure 2C–D).

**Figure 4:**
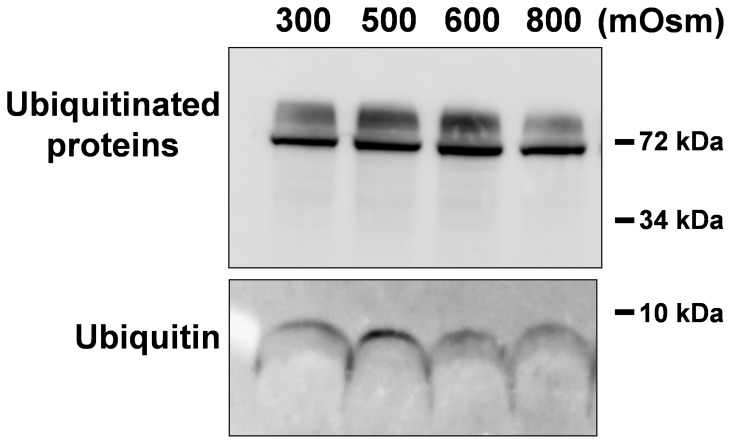
Increased osmolarity does not change the protein level of endogenous ubiquitin. BHK clone 19 myc-CLN3-expressing cells were grown under isotonic (300 mOsm) or hyperosmotic conditions. Osmolarity was increased at 100 mOsm intervals to 500, 600 or 800 mOsm by the addition of NaCl plus urea (1.5∶1 molar ratio). After being exposed to 500, 600 or 800 mOsm for 24 hours, cell lysates were prepared using 1% DDM detergent under non-denaturing conditions. Twenty-µg protein from each sample was loaded on a 16.5% Tris-Tricine polyacrylamide gel and immunoblotted with a mouse monoclonal anti-ubiquitin antibody. Increasing osmolarity did not change the expression level of free ubiqutin (8 kDa) or ubiquitinated proteins (∼70 kDa). Immunoblot representative of 3 biological replicates is shown.

**Figure 5:**
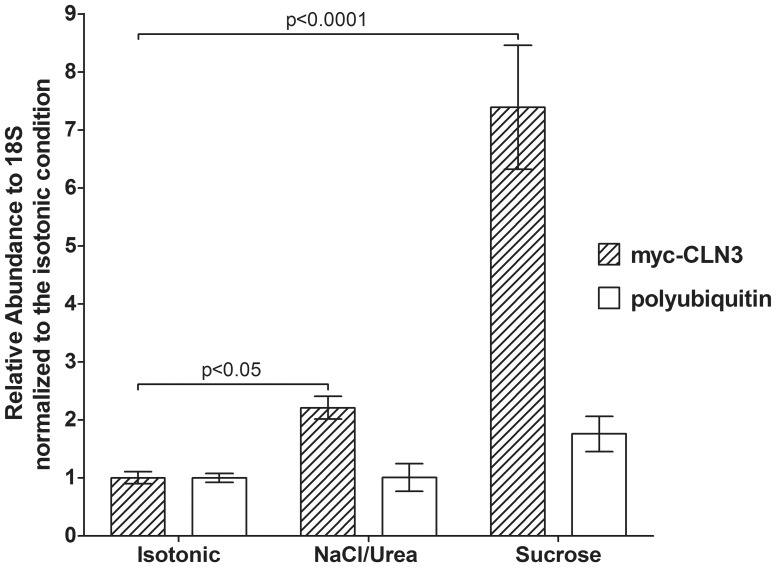
Increased osmolarity upregulates the mRNA level of ***myc-CLN3*** **.** BHK clone 19 myc-CLN3-expressing cells were grown under isotonic (300 mOsm) or hyperosmotic conditions. Osmolarity was increased at 100 mOsm intervals to 800 mOsm by the addition of either NaCl/urea or sucrose. After being exposed to 800 mOsm for 24 hours, mRNA levels of *myc-CLN3* and *polyubiquitin* were determined by quantitative real-time RT-PCR, normalized to the level of 18S ribosomal RNA, and expressed as fold increase of the isotonic control. Columns and bars represent mean ± S.E.M. (n = 3–6). Statistical significance was determined by one-way ANOVA with Bonferroni’s post-test.

BHK clone 19 cells express myc-CLN3 under the control of the human ubiquitin C promoter. In order to determine if the osmotic stress-induced increase in myc-CLN3 protein expression is due to an upregulation of the ubiquitin promoter, we examined if the protein level of endogenous ubiquitin changes with increasing osmolarity. Ubiquitin immunoblotting revealed that hyperosmolarity did not change the expression level of free ubiqutin (8 kDa) or ubiquitinated proteins (Figure 4). We also measured the mRNA level of the endogenous *polyubiquitin* gene and found that hyperosmolarity does not increase the *polyubiquitin* transcript level in cells stably expressing myc-CLN3 (Figure 5). However, when *myc-CLN3* mRNA expression was measured using primers specific for the *myc-CLN3* junction, it was apparent that the *myc-CLN3* transcript is significantly increased in hyperosmolarity (Figure 5).

### Osmotic stress changes the subcellular localization of CLN3

**Figure 6:**
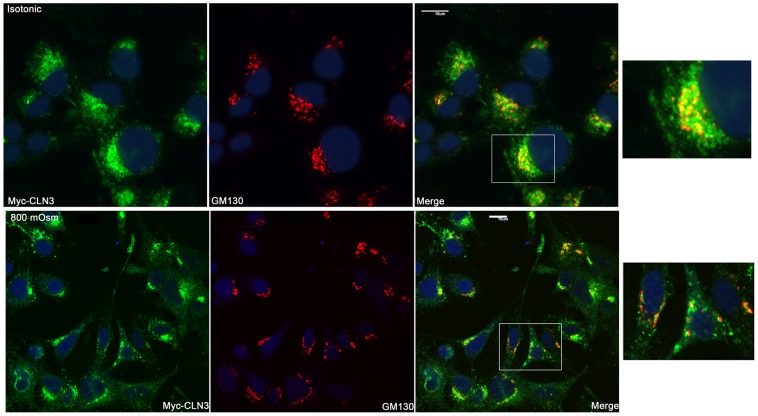
Osmotic stress decreases the Golgi localization of myc-CLN3. BHK clone 19 myc-CLN3-expressing cells were grown on poly-D-lysine coated coverslips either under isotonic (300 mOsm) or hyperosmotic conditions. Osmolarity was increased at 100 mOsm intervals to 800 mOsm by the addition of NaCl/urea. After being exposed to 800 mOsm for 24 hours, cells were fixed, permeabilized and immunofluorescently stained for the myc-tag (to detect myc-CLN3; green) and for the Golgi marker, GM130 (red). Under isotonic conditions myc-CLN3 is primarily in the perinuclear region with prominent Golgi localization. In the merged images yellow indicate the Golgi-localized myc-CLN3. Osmotic stress decreases the Golgi localization of myc-CLN3, and CLN3 appears in the whole cell body and in cellular processes. The white-framed areas of the merged images are enlarged at the right side of the figure to highlight the osmotic stress-induced changes in myc-CLN3 localization. Images were acquired with a confocal microscope. Scale bars indicate 10 µm.

**Figure 7:**
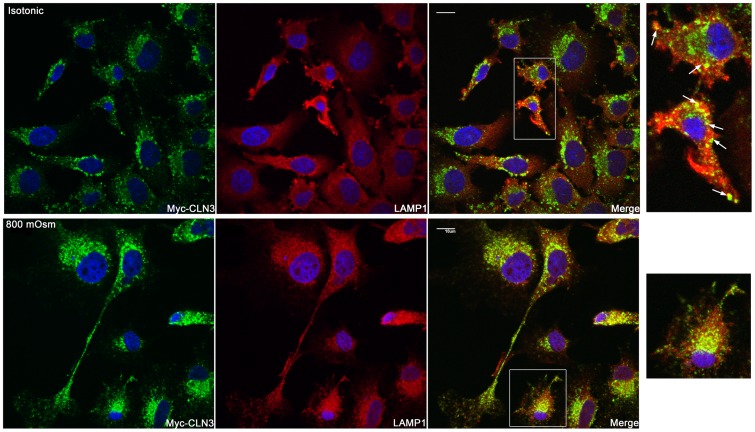
Osmotic stress enhances the lysosomal localization of myc-CLN3. BHK clone 19 myc-CLN3-expressing cells were grown on poly-D-lysine coated coverslips either under isotonic (300 mOsm) or hyperosmotic conditions. Osmolarity was increased at 100 mOsm intervals to 800 mOsm by the addition of NaCl/urea. After being exposed to 800 mOsm for 24 hours, cells were fixed, permeabilized and immunofluorescently stained for the myc-tag (to detect myc-CLN3; green) and for the lysosomal marker, LAMP1 (red). Under isotonic conditions, a portion of myc-CLN3 is localized to lysosomes. In the merged images yellow indicates the lysosomal localization of myc-CLN3. Myc-CLN3 distribution in lysosomes is enriched at 800 mOsm. The white-framed areas of the merged images are enlarged at the right side of the figure to highlight the osmotic stress-induced increase in the lysosomal localization of myc-CLN3. Arrows in the enlarged images taken from isotonic cultures point to myc-CLN3 localized in lysosomes. Images were taken with a confocal microscope. Scale bars indicate 10 µm.

**Figure 8:**
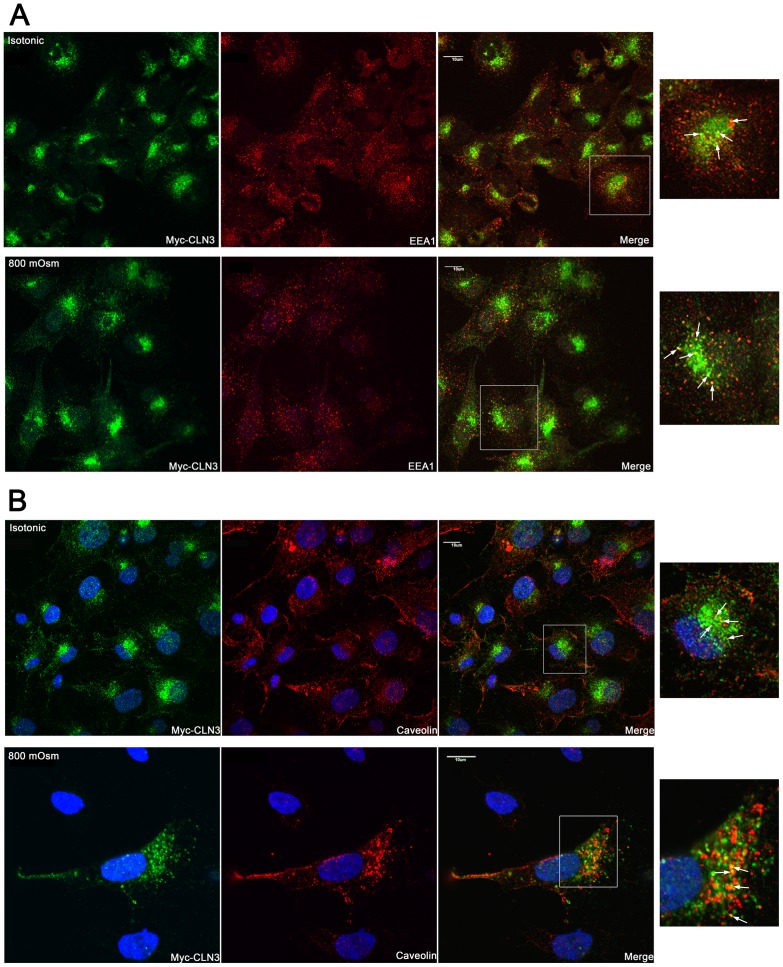
Under both isotonic and hyperosmotic conditions, a portion of myc-CLN3 is localized in early endosomes and in cholesterol/sphingolipid-enriched plasma membrane microdomain caveolae. BHK clone 19 myc-CLN3-expressing cells were grown on poly-D-lysine coated coverslips either under isotonic (300 mOsm) or hyperosmotic conditions. Osmolarity was increased at 100 mOsm intervals to 800 mOsm by the addition of NaCl/urea. After being exposed to 800 mOsm for 24 hours, cells were fixed, permeabilized and immunofluorescently stained for the myc-tag (to detect myc-CLN3; green) and for either the early endosomal marker, EEA1 (A) or the caveolae marker, caveolin 1 (B). In the merged images yellow indicates the colocalization of myc-CLN3 with EEA1 (A) or caveolin (B). The white-framed areas of the merged images are enlarged at the right side of the figure to highlight the early endosomal and caveolar localization of myc-CLN3. Arrows in the enlarged images point to colocalizations. Images were taken with a confocal microscope. Scale bars indicate 10 µm.

Using immunofluorescent staining and confocal microscopy the subcellular localization of myc-CLN3 was analyzed in BHK clone 19 myc-CLN3-expressing cells grown either under isotonic (300 mOsm) or hyperosmotic (800 mOsm) conditions. Under isotonic conditions, myc-CLN3 was found in a punctate vesicular pattern surrounding the nucleus with prominent Golgi (Figure 6) and lysosomal (Figure 7) localizations. The large CLN3-positive vesicles, which can be seen in both Figures 6 and 7, are most noticeable in the first image of Figure 7. We also observed CLN3-positive early endosomes (Figure 8), late endosomes (Figure S1) and cholesterol/sphingolipid-enriched plasma membrane microdomain caveolae (Figure 8). Myc-CLN3 did not colocalize with the endoplasmic reticulum (ER) protein GRP78, the tight junction protein, ZO-1or with β-catenin (data not shown).

When the osmolarity of the culture medium was increased to 800 mOsm by addition of a combination of NaCl and urea (1.5:1), BHK cells became more elongated and less tightly packed. Osmotic stress decreased the Golgi localization of myc-CLN3 and CLN3 appeared in the whole cell body and in cellular processes (Figure 6). Osmotic stress also affected the lysosomal localization of myc-CLN3, significantly enhancing it (Figure 7). While at isotonic conditions (300 mOsm) 21±1.4% of myc-CLN3 colocalized with the lysosomal marker LAMP1, osmotic stress (800 mOsm) increased this colocalization to 45±5.6% (p<0.0001 by unpaired t-test). The increased osmolarity did not cause obvious changes in the partial early endosomal and caveolar localization of myc-CLN3 (Figure 8).

## Discussion

The yeast orthologue of CLN3, Btn1p, has been localized to the Golgi as well as the vacuole (the analogous structure to the mammalian lysosome) in two yeast species, *Saccharomyces cerevisiae* [29], [30] and *Schizosaccharomyces pombe* [31]. In mammalian cells, CLN3 has been localized to different membranes in many different studies that used CLN3-overexpressing vectors and/or anti-CLN3 antibodies with questionable specificity. High overexpression of membrane proteins could saturate trafficking or post-translational processing complexes, producing artifactual observations that may not be relevant for the biology of the protein. In studying CLN3, exogenous expression is required due to the unavailability of useful antibodies to detect endogenous CLN3. Also, the endogenous promoter of CLN3 has not been identified and characterized. Many studies use the elongation factor 1-α (EF1-α) or cytomegalovirus (CMV) promoters to drive exogenous expression of target proteins. Expression by these promoters is often saturating, and in some cases, unstable by cell silencing mechanisms due to the high overexpression [32]. In this study, the human ubiquitin C promoter was chosen to drive CLN3 expression at a moderate level [25]. In order to detect CLN3 in the absence of antibodies that detect endogenous CLN3, a small myc epitope tag was added to the N-terminus. Excellent antibodies have been generated in rabbit and mouse and are commercially available for myc, and therefore these antibodies can be used in many applications to study myc-tagged CLN3. The N-terminus of CLN3 was tagged as there are several protein interactors of CLN3 that bind to the C-terminus [33]. Additionally, the C-terminus of CLN3 is farnesylated, which is important for its localization [8].

To study the conditional expression and subcellular localization of CLN3 we generated a stably transfected BHK clonal cell line that expresses a moderate level of myc-tagged human CLN3 under the control of the human ubiquitin C promoter. Under isotonic conditions human myc-CLN3 was found in a punctate vesicular pattern surrounding the nucleus with prominent Golgi and lysosomal localizations. CLN3-positive early endosomes, late endosomes and cholesterol/sphingolipid-enriched plasma membrane microdomain caveolae were also observed. Increasing the osmolarity of the culture medium to 800 mOsm extended CLN3 distribution away from the perinuclear region and enhanced the lysosomal localization of CLN3. Currently we do not know if the change in CLN3 subcellular distribution is a direct response to the increased osmolarity or just a consequence of the increased CLN3 expression. Finding an alternative way to induce increased CLN3 levels will help to resolve this issue. Although, we detected a portion of myc-CLN3 in early endosomes, late endosomes and caveolae, the punctate structure that myc-CLN3 is found in outside of the Golgi and lysosomes is not totally defined and may be dynamic. This coincides with recently published results of the yeast homolog of CLN3, Btn1p. Btn1p was found in the vacuole, the analogous structure to the mammalian lysosome, but Btn1p was also found in punctate spots that did not colocalize with several known vesicular markers [30]. Though the mechanism of CLN3 distribution to the periphery of the cell in hyperosmotic conditions is not yet well understood, it does suggest that CLN3 localization is responsive to cellular conditions in mammalian cells, as it was shown in yeast [30].

In yeast, expression of *Btn1,* the homolog to *CLN3*, increases as the growth medium of cells is changed from pH 4 to pH 6. Yeast cells growing at pH 6 have to work harder to acidify the medium than cells at pH 4, so it would seem that Btn1p is required at the higher pH [30]. Conditional regulation of mouse *Cln3* expression has also been reported. Stein et al. (2010) showed that increasing osmolarity in mouse primary kidney cell cultures upregulated *Cln3* mRNA expression. In our study presented here, osmotic stress is observed to cause an increase in human *CLN3* mRNA and protein levels. The regulatory mechanism of this upregulation, however, may be different from that behind the increased mouse *Cln3* expression in the study by Stein et al. (2010) because the endogenous *CLN3* promoter was not involved in our experiments, *CLN3* was expressed under the control of the human ubiquitin C promoter. Osmoregulation of the human ubiquitin C promoter has not been reported, and we have evidence showing that during hyperosmolarity neither the endogenous ubiquitin protein level (Figure 4) nor the endogenous *polyubiquitin* transcript (Figure 5) is elevated. Furthermore, NIH BLAST search revealed that the human ubiquitin C promoter does not contain any of the 3 human osmoregulatory elements [34]. The possibility that the site within the genome where the *myc-CLN3*-expressing plasmid has stably inserted is sensitive to osmoregulation cannot be ruled out. Even if this happened, *myc-CLN3* expression still remained under the control of the human ubiquitin C promoter, which is not osmoregulated. Based on the above, the increased *myc-CLN3* mRNA level must be a result of increased mRNA stability and/or enhancer element(s) in *CLN3*.

A search for transcription factor binding sites in the cDNA of *CLN3* using TFSEARCH (http://www.cbrc.jp/research/db/TFSEARCH.html) [35]–[40] with a threshold score of 96 (maximum score is 100) identified 14 potential binding sites for 7 transcription factors: USF [41], GATA-1 [42], SREBPs [43], HSF1 and HSF2 [44], [45] binding sites in the 5’ untranslated region, and AML-1a [46], HSF2 and AP-1 [47] binding sites in the coding region. Two of these transcription factors, AP-1 and HSF1 are known to be activated by osmotic stress [48], [49], and they may be responsible for the observed upregulation of *myc-CLN3* mRNA in our study. Though the presence of transcription factor binding sites in exonic sequences (in the cDNA of *CLN3*) seems unusual, it was recently discovered that exons in human genes contain a number of transcriptional regulatory elements that are enriched in transcription factor binding sites [50].

Under hyperosmotic conditions, there is a decrease in cell volume, which induces crowding of proteins and potentially protein aggregation [51], [52]. This process may also have an effect on CLN3 localization. After the initial decrease in cell volume the cell cycle arrests briefly to allow the cells to adapt [53]. The localization of certain proteins has been shown to also change in response to osmotic stress. For example, myosin IIB has been shown to be rapidly translocated from the cytosol to the cortical region, beneath the plasma membrane [54]. Since CLN3 interacts with several proteins [33] including myosin IIB [55], it is possible that the observed alteration in CLN3 localization under increased osmolarity is a downstream result of changes in the location of proteins directly or indirectly interacting with CLN3.

Numerous protein levels change in response to hyperosmotic stress [53]. A separate study using a proteomic screen of cells treated in both hypo- and hyper-osmotic conditions revealed numerous alterations in protein levels, including an upregulation of protein degradation under hypo-osmotic stress, but not under hyperosmotic stress [56]. Lysosomal proteins in general do not seem to be represented in these types of proteomic screens, so an increase in myc-CLN3 does not appear to be simply a nonspecific increase in lysosomal biogenesis under these conditions.

The results of our study suggest that CLN3 is involved in sensing osmotic stress or responsive to it by ensuring that cytoskeleton and appropriate receptors or channels are positioned at particular membranes in order to deal with the structural and ionic stress of adapting to hyperosmolarity. CLN3 interacts with the plasma membrane Na^+^-K^+^-ATPase [28], the membrane-associated cytoskeletal protein, fodrin [28] and nonmuscle myosin IIB [55], and these interactions may play a role in the adaptation to hyperosmolarity.

In the present study we showed that CLN3 has multiple subcellular localizations within the cell, which, together with its expression, prominently change following osmotic stress. These data suggest that CLN3 is involved in the response and adaptation to cellular stress.

## Materials and Methods

### Plasmid construction

Human *CLN3* cDNA (GenBank: NM_001042432.1) was cloned in-frame into the *Xho1* and *Age1* restriction sites of the pUB6-B vector (Life Technologies, Grand Island, NY). The N-terminal myc-tag was added using primer based methods. Confirmation of proper ligation was completed using diagnostic restriction enzyme analysis using *HindIII* and DNA sequencing. All restriction enzymes were obtained from New England Biolabs (Ipswich, MA).

### Cell Culture

BHK cells were obtained from the American Type Culture Collection (ATCC, Manassas, VA). BHK cells were cultured in high glucose Dulbecco’s Modified Eagle’s Medium (DMEM), 10% fetal bovine serum (FBS), MEM non-essential amino acids (NEAA) (100 µM), penicillin-streptomycin (100 U/ml/100 µg/ml) (all from Hyclone, ThermoFisher Scientific, Waltham, MA), and 1X GlutaMax (Life Technologies, Grand Island, NY). BHK cells were transfected with the UB6-myc-CLN3 construct or empty UB6 vector using Lipofectamine 2000 (Life Technologies, Grand Island, NY) according to the manufacturer’s protocol. Twenty-four hours after the transfection the cells were treated with trypsin and sorted into 48-well plates at a concentration of 1 cell/well. Following a 6-hour incubation to allow the cells to settle and adhere to the bottom of the wells, the medium was replaced with blasticidin-containing complete medium, which was replenished every two days for 2 weeks. Clones that were isolated from these plates were then expanded into 24-well, 12-well, and 6-well plates prior to screening for myc-CLN3 expression. Any clones that were re-derived from liquid nitrogen storage were grown under blasticidin (50 µg/ml) selection for a minimum of 2 weeks before use in any experiments. No cells were used beyond passage 15, or approximately 8 weeks in culture, to prevent genetic drift or contamination of the cultures.

### Cell culture under hyperosmolarity conditions

Cells in complete, blasticidin-containing medium were plated in 10-cm culture dishes (50,000 cells/dish) for immunoblot and RT-PCR analyses, and on poly-D-lysine (10 µg/ml) coated coverslips in 24-well plates (1,700 cells/well) for immunofluorescent staining. Cells were allowed to adhere overnight. Each subsequent day, the growth medium was replaced with complete medium (isotonic, 300 mOsm) supplemented with increasing osmolarity. Osmolarity was increased at 100 mOsm intervals to 500 mOsm, 600 mOsm, or 800 mOsm by the addition of NaCl plus urea (1.5∶1 molar ratio). Sucrose was added similarly when indicated.

### RT-PCR

To identify *myc-CLN3*-expressing BHK clones, clones were grown in 10-cm culture dishes under blasticidin selection and RNA was obtained using standard Trizol extraction (Life Technologies, Grand Island, NY). In brief, cells were washed three times with ice-cold Hank’s Balanced Salt Solution (HBSS) and then Trizol reagent was added. Cells were scraped from the plates and incubated with Trizol to extract and fractionate nucleic acids. RNA was precipitated using isopropyl alcohol and isolated by centrifugation. These RNA samples were then treated with reverse-transcriptase to generate cDNA. Aliquots of RNA samples were treated similarly without reverse-transcriptase added, to provide a negative control that would indicate genomic DNA contamination. The cDNA was then tested for *myc-CLN3* expression using primers specific for the *myc-CLN3* junction, which will exclude endogenous *Cln3*. Forward and reverse primers for the *myc-CLN3* junction were CTGAACTTGATGCGATGGAACAAAA and CCCCTCGGAATCCGAAAAGC, respectively. Species-specific *GAPDH* was used as a control.

To quantify *myc-CLN3* and *polyubiquitin* mRNA levels, BHK clone 19 myc-CLN3 expressing and BHK clone 2 UB6 empty vector expressing cells were grown in blasticidin-containing medium, in 10-cm culture dishes under isotonic (300 mOsm) or hyperosmotic conditions. Osmolarity was increased at 100 mOsm intervals to 800 mOsm by the addition of either NaCl/urea or sucrose. Cells were exposed to 800 mOsm for 24 hours. RNA was extracted from cells harvested after growing an equal amount of time under isotonic or hyperosmolarity conditions using PerfectPure RNA Cultured Cell Kit (5’Prime) and Maxwell 16 LEV Simply RNA Cells Kit (Promega, Madison, WI). Biological triplicates were grown for isotonic and hyperosmolarity conditions. All extracted RNA samples were run on a BioAnalyzer (Agilent, Santa Clara, CA) to determine integrity. cDNA was generated using High Capacity Reverse Transcription Kit (Life Technologies, Grand Island, NY) from 2 µg of RNA for each sample on a BioRad ThermoCycler. The resulting cDNA was then tested for *myc-CLN3* (using primers specifically designed for the *myc-CLN3* junction, which excludes endogenous *Cln3*; see above), *polyubiquitin* and 18S rRNA expression. Forward and reverse primers for the hamster *polyubiquitin* were TGCAGATCTTTGTGAAGA and CCTTGACATTCTCGATGG, respectively.

Quantitative PCR was set up using Absolute Blue QPCR Master Mix (ThermoFisher Scientific, Waltham, MA) and carried out on a Stratagene Mx3005P QPCR system. *Polyubiquitin* and *myc-CLN3* mRNA levels were normalized to 18S rRNA expression.

### Antibodies

The rabbit (2272) and mouse (2276) anti-myc-tag antibodies were obtained from Cell Signaling Technologies (Danvers, MA). The mouse anti-myosin IIB antibody (CMII 23) was purchased from the Iowa Developmental Studies Hybridoma Bank, (NICHD, University of Iowa, Iowa City, IA). The mouse anti-ubiquitin (05-944) antibody was from EMD Millipore (Billerica, MA). Rat monoclonal anti-LAMP1 antibody (sc-19992) was obtained from Santa Cruz Biotechnology (Santa Cruz, CA). Mouse antibodies against GM130 (610822), EEA1 (610456) and caveolin 1 (610406) were purchased from BD Biosciences (San Jose, CA). Alexa Fluor 488- and 594-conjugated secondary antibodies and DAPI nuclear stain were acquired from Life Technologies (Grand Island, NY).

### Immunoblotting

Protein samples were prepared from equal numbers of cells grown in 10-cm culture dishes (Corning Inc., Corning, NY). Cells were washed three times with ice-cold PBS and then scraped into 1 ml of PBS, followed by centrifugation at 200 g. Cell pellets were treated for 30 minutes with non-denaturing lysis buffers containing either Triton X-100 (50 mM Tris-HCl, pH 7.5, 300 mM NaCl, 5 mM EDTA, 1% Triton X-100, pH 7.4) or n-Dodecyl β-D-maltoside DDM) (1% DDM, 50 mM sodium phosphate buffer, pH 7.4) to extract proteins on ice. Following a high speed spin (>14,000 g), the post-nuclear supernatant was collected, and the protein concentration was measured using the Pierce 660 nm Protein Assay (ThermoFisher Scientific, Waltham, MA).

Samples were heated in Laemmli buffer at 37°C for 15 minutes when looking to detect myc-CLN3 and GM130, or at 100°C for 5 minutes for ubiquitin. Proteins were resolved on 10 or 12% polyacrylamide gels. To detect free ubiquitin (8 kDa) 16.5% Tris-Tricine polyacrylamide gels combined with Tris-Tricine buffer were used. After SDS-PAGE, proteins were transferred to PVDF or nitrocellulose membranes (Millipore, Billerica, MA) using the standard wet transfer method at 100V for 90 minutes. Membranes were then incubated in blocking buffer [5% milk in 100 mM Tris-HCl, pH 7.5, 150 mM NaCl, 0.1% Tween-20 (TBST)] for 1 hour. Primary antibodies were applied in blocking buffer overnight at 4°C. Membranes were washed 3 times, for 5 minutes each with TBST, and incubated with a secondary antibody conjugated with horseradish peroxidase (GE Healthcare) in blocking buffer for 1 hour at room temperature. After washing the membranes with TBST 4 times (5 minutes each), signal was detected by chemiluminescence using ECL Plus Western Blotting Detection kit (GE Healthcare Life Sciences, Piscataway, NJ) and a BioSpectrum UVP Imaging system (Upland, CA).

### Immunofluorescence

Cells were grown on poly-D-lysine (10 µg/ml) coated coverslips. Cells were fixed (3% paraformaldehyde, 3% sucrose in PBS), permeabilized [0.1% Triton-X in TBS (100 mM Tris-HCl, pH 7.5, 150 mM NaCl)] and blocked with Blotto (4% milk in TBS, 0.1% Triton X-100) for 1 hour prior to adding primary antibodies in Blotto, as indicated, overnight at 4°C. After washing with Blotto or TBS-0.01% Triton-X, compatible Alexa Fluor 488- and 594-conjugated secondary antibodies were applied for one hour at room temperature and then washed using TBS-0.1% Triton X-100. Cells were mounted in ProLong Anti-Fade mounting medium (Life Technologies) and sealed with clear nail polish.

For LAMP1 detection by immunofluorescence, cells were fixed with 4% paraformaldehyde and permeabilized with 0.02% saponin in TBS. The blocking buffer used contained 4% milk and 0.02% saponin in TBS, and all washes were completed with either saponin-containing blocking buffer or 0.02% saponin in TBS. No Triton-X-100 containing buffer can be used when detecting LAMP1 by immunocytochemical staining. Rat anti-LAMP1 and anti-rat secondary Alexa Fluor-488-conjugated antibodies were incubated in saponin-containing blocking buffer.

Images were acquired using an inverted Olympus IX81 and Olympus FluoView FV1000 confocal microscope (Olympus America, Inc; Center Valley, PA) and the Olympus FluoView Ver. 2.6c software or a Nikon Eclipse 90i confocal microscope (Nikon instruments, Inc., Melville, NY) with a CoolSNAP HQ camera (Roper Scientific GmbH, Germany) and NIS-Elements software package.

Quantification of myc-CLN3 and LAMP1 colocalization as percentage of total myc-CLN3-positive immunostaining was carried out using the Colocalization plugin of the NIH ImageJ program. Confocal images of 35 control (300 mOsm) cells and 27 osmotically stressed (800 mOsm) cells were analyzed; mean ± SD values are reported in the Results.

## Supporting Information

Figure S1 **A fraction of myc-CLN3 is localized to late endosomes.** BHK clone 19 myc-CLN3 expressing cells were grown under isotonic (300 mOsm) conditions. Late endosomes were pulse-chase labeled according to a method developed to label late endosomes in BHK cells [57]. Cells grown on poly-D-lysine coated coverslips were incubated with Alexa Fluor 488-labeled dextran (Life Technologies) for 5 min at 37°C. After washing twice with culture medium, cells were incubated in dextran-free culture medium for 40 min at 37°C to label late endosomes (green). At the end of the 40-min incubation cells were fixed, permeabilized and immunofluorescently stained for the myc-tag (to detect myc-CLN3; red, Alexa Fluor 594). In the merged image yellow indicates the colocalization of myc-CLN3 with late endosomes; arrows point to colocalizations. Images were taken with a confocal microscope. Scale bars indicate 10 µm.
(TIF)
