# Supplementary figures and images for: Osmotic Stress Changes the Expression and Subcellular Localization of the Batten Disease Protein CLN3
Source: PLoS One. 2013 Jun 20;8(6):e66203. doi: 10.1371/journal.pone.0066203 (PMC3688782; doi:10.1371/journal.pone.0066203)

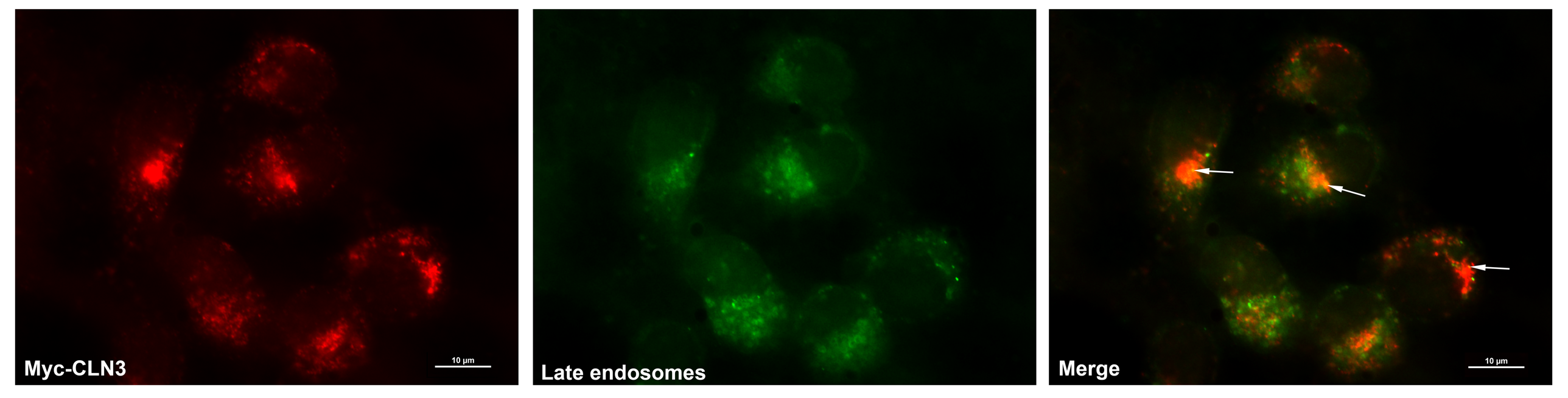

Supplement: Figure S1 — A fraction of myc-CLN3 is localized to late endosomes. BHK clone 19 myc-CLN3 expressing cells were grown under isotonic (300 mOsm) conditions. Late endosomes were pulse-chase labeled according to a method developed to label late endosomes in BHK cells [57]. Cells grown on poly-D-lysine coated coverslips were incubated with Alexa Fluor 488-labeled dextran (Life Technologies) for 5 min at 37°C. After washing twice with culture medium, cells were incubated in dextran-free culture medium for 40 min at 37°C to label late endosomes (green). At the end of the 40-min incubation cells were fixed, permeabilized and immunofluorescently stained for the myc-tag (to detect myc-CLN3; red, Alexa Fluor 594). In the merged image yellow indicates the colocalization of myc-CLN3 with late endosomes; arrows point to colocalizations. Images were taken with a confocal microscope. Scale bars indicate 10 µm. (TIF) [file pone.0066203.s001.tif]
